# Conditioned medium from human tonsil-derived mesenchymal stem cells inhibits glucocorticoid-induced adipocyte differentiation

**DOI:** 10.1371/journal.pone.0266857

**Published:** 2022-06-01

**Authors:** Yu-Hee Kim, Hyun-Ji Lee, Kyung-Ah Cho, So-Youn Woo, Kyung-Ha Ryu

**Affiliations:** 1 Department of Microbiology, Ewha Womans University College of Medicine, Gangseo-Gu, Seoul, South Korea; 2 Advanced Biomedical Research Institute, Ewha Womans University Seoul Hospital, Gangseo-Gu, Seoul, South Korea; 3 Department of Pediatrics, Ewha Womans University College of Medicine, Gangseo-Gu, Seoul, South Korea; Università degli Studi della Campania, ITALY

## Abstract

Obesity, which has become a major global health problem, involves a constitutive increase in adipocyte differentiation signaling. Previous studies show that mesenchymal stem cells (MSCs) induce weight loss and glycemic control. However, the mechanisms by which MSCs regulate adipocyte differentiation are not yet known. In this study, we investigated the effects of conditioned medium obtained from human tonsil-derived MSCs (T-MSC CM) on adipocyte differentiation. We found that T-MSC CM attenuated adipocyte differentiation from early stages via inhibiting glucocorticoid signaling. T-MSC CM also increased the phosphorylation of p38 mitogen-activated protein kinase and glucocorticoid receptors and decreased the subsequent nucleus translocation of glucocorticoid receptors. Chronic treatment of mice with synthetic glucocorticoids induced visceral and bone marrow adipose tissue expansion, but these effects were not observed in mice injected with T-MSC CM. Furthermore, T-MSC CM injection protected against reductions in blood platelet counts induced by chronic glucocorticoid treatment, and enhanced megakaryocyte differentiation was also observed. Collectively, these results demonstrate that T-MSC CM exerts inhibitory effects on adipocyte differentiation by regulating glucocorticoid signal transduction. These findings suggest that the therapeutic application of T-MSC CM could reduce obesity by preventing adipose tissue expansion.

## Introduction

Obesity has become a global problem, with a near tripling of the worldwide prevalence of obesity between 1975 and 2016 according to the World Health Organization. Obesity is accompanied by many chronic diseases such as cardiovascular disease, diabetes, musculoskeletal disorders, and some cancers [1], for which the presence of obesity further increases morbidity and mortality [2]. In the cases of cancers, for example, it is shown that adipose tissue-derived mesenchymal stem cells (Ad-MSCs) promote tumor progression and metastasis [3], and obesity exaggerates these deleterious effects *in vitro* and *in vivo* [4]. Another study demonstrated that diet-induced TAZ expression and increased secretion of resistin support the enhanced tumorigenesis in obesity [5]. Therefore, therapeutic strategies for reducing obesity are required to lessen its burden on healthcare systems around the globe [6].

Obesity develops as a result of adipocyte hypertrophy and hyperplasia. Surplus calories are stored inside adipocytes in the form of lipid droplets that increase the size of cells, which release signals that induce the proliferation and differentiation of preadipocytes. Signals that activate adipocyte differentiation include insulin, glucocorticoids (GCs), and phosphodiesterase inhibitors, which have independent mechanisms of actions [7]. In particular, GC signaling regulates the initial phase of differentiation as evidenced by limited adipocyte differentiation capacity in the absence of GCs [8]. Once adipocyte differentiation is primed by GCs, phosphodiesterase inhibitors maintain the adipogenic pathway via stimulation of cAMP signaling. Adipocyte differentiation is then completed by lipid droplet formation through the action of insulin. Excessive GCs in patients with Cushing’s syndrome are linked to abdominal obesity [9]. Also, repeated injection of insulin in a patient with type 2 diabetes increased adipogenesis in the injection area [10]. Therefore, targeting the adipocyte differentiation pathway could be a strategy for combating obesity-associated diseases.

Previous studies investigated the application of mesenchymal stem cells (MSCs) for the treatment of obesity and related diseases. Injection of Ad-MSCs reduces body weight and blood glucose levels in high-fat diet-induced obese mice [11] and ameliorates hyperglycemia and insulin resistance in a rat model of type 2 diabetes [12,13]. Likewise, our research group found that injecting MSCs isolated from human palatine tonsils (T-MSCs) reduces high-fat diet-induced weight gain in mice [14]. We also found that conditioned medium (CM) from T-MSCs inhibits adipocyte differentiation and reduces weight gain in senescence-accelerated mouse prone 6 (SAMP6) mice, which exhibit obesity in aging [15,16]. To extend these previous observations, we aimed to elucidate the mechanisms by which T-MSC CM inhibits adipocyte differentiation.

## Materials and methods

Unless otherwise stated, general reagents were from Merck KGaA (Darmstadt, Germany), and cell culture reagents were from Welgene (Gyeongsan, South Korea).

### T-MSC culture and preparation of CM

Previously isolated and characterized T-MSCs were cultured in low-glucose Dulbecco’s Modified Eagle Medium (DMEM) supplemented with 10% fetal bovine serum (FBS) and antibiotics (100 IU/mL penicillin and 100 μg/mL streptomycin; Capricorn Scientific, Ebsdorfergrund, Germany). T-MSCs passage 7–9 were seeded at concentrations of 0.2–0.5 × 10^4^/mL and culture medium was replaced every 3–4 days until cells reached 80% confluency, after which medium was changed and incubated for an additional 48 h for CM collection. CM was concentrated 20-fold using an Amicon Ultra centrifugal filter unit (EMD Millipore, Darmstadt, Germany) by centrifugation at 5000 rpm for 1 h at 4°C.

### Flow cytometry

To determine the expression of mesenchymal stem cell markers on T-MSCs, cells were collected and pelleted by centrifugation at 1300 rpm for 5 min. After washing with PBS, cells were pelleted again and stained with FITC-conjugated anti-human CD11b, PE-conjugated anti-human CD34, PerCP-conjugated anti-human CD45, APC-conjugated anti-human CD73, PE-conjugated anti-human CD90, or PE-conjugated anti-human CD105 antibody (Biolegend, San Diego, CA, USA) diluted in FACS buffer (PBS supplemented with 10% FBS, 10 mM EDTA, 20 mM HEPES, and 1 mM sodium pyruvate) for 30 min on ice. To determine the expression of megakaryocytic marker on differentiated K562 cells, Pacific Blue-conjugated anti-human CD41 antibody (Biolegend) was used. After washing cells with buffer, surface protein expression was measured using a NovoCyte flow cytometer (ACEA Biosciences, San Diego, CA, USA) and analyzed using NovoExpress software.

### Adipocyte differentiation

The 3T3-L1 mouse preadipocyte cell line, purchased from the Korean Cell Line Bank, was maintained in high-glucose DMEM supplemented with 10% FBS and antibiotics. After reaching confluency, the medium was changed, and cells were incubated for 3 more days. For induction of adipocyte differentiation, 4 mg/mL insulin, 0.25 μM dexamethasone, and 0.5 mM 3-isobutyl-1-methylxanthin (IBMX) were added to the culture medium for the first 3 days, and insulin was added for the next 2 days. OP9 mouse bone marrow stromal cells (ATCC, Manassas, VA, USA) were cultured in Alpha Minimum Essential Medium without ribonucleosides and deoxyribonucleosides (Thermo Fisher Scientific, Waltham, MA, USA) supplemented with 20% FBS and antibiotics until reaching confluency. Cells were then induced for adipocyte differentiation with the differentiation medium used for 3T3-L1 cells.

### Oil Red O staining

Lipid accumulation was measured using Oil Red O staining. On differentiation day 7, cells were rinsed with PBS and fixed in 10% formalin for 5 min at room temperature. Oil Red O stock solution (8.5 mM) prepared in isopropanol was mixed with distilled water at a 6:4 ratio and added to cells for 10 min. After washing the wells with distilled water, the stain was eluted with isopropanol, and absorbance was measured at 540 nm.

### Adipogenesis PCR array

RNA was harvested from 3T3-L1 adipocytes on differentiation day 3 using a NucleoSpin RNA Kit (Macherey-Nagel, Duren, Germany), cDNA was synthesized using a RT^2^ First Strand Kit (Qiagen, Hilden, Germany), and polymerase chain reaction (PCR) array was performed on a QuantStudio 3 Real-Time PCR machine (Thermo.Fisher Scientific) using the RT^2^ Profiler PCR Array for Mouse Adipogenesis (Qiagen) according to the manufacturer’s instructions. Results were analyzed using RT^2^ Profiler PCR Array Data Analysis v3.5 (http://pcrdataanalysis.sabiosciences.com/pcr/arrayanalysis.php).

### Real-Time PCR

RNA extraction was performed using TRIzol (Thermo Fisher Scientific), and cDNA was synthesized using Reverse Transcription Master Premix (ELPIS-Biotech, Daejeon, Korea). Real-time PCR was performed using a SensiFAST SYBR Hi-ROX kit (Bioline, London, UK) on a StepOnePlus Real-Time PCR machine (Thermo Fisher Scientific). Gene amplification was conducted using 40 cycles of a 15-s denaturation step at 95°C and a 1-min amplification and signal acquisition step at 62°C. Cyclophilin A was used as a housekeeping gene, and relative expression of the target gene was determined as 2^(Ct cyclophilin A–Ct target gene)^. Primer sequences are listed in Table 1.

**Table 1:** Sequences of primers used for real-time quantitative PCR.

Gene	GeneBank accession	Primer sequence	
		Forward (5’– 3’)	Reverse (5’– 3’)
Cyclophilin	NM_021130.4	CGTTTTGGGTCCAGGAATGG	TACAGGACATTGCGAGCAGA
Adig	NM_145635.2	CAGGTACCATCTGGGCCTAA	GCTCTGTAGGGGCACAAGAG
Adipoq	NM_009605.4	GTTCCCAATGTACCCATTCGC	TGTTGCAGTAGAACTTGCCAG
Agt	NM_007428.3	TCCCGACTAGATGGACACAAG	AGAGGGCAGGGGTAAAGAGAG
Cfd	NM_013459.4	TACATGGCTTCCGTGCAAGTG	CACAGAGTCGTCATCCGTCA
Cebpa	NM_007678.3	CAAGAACAGCAACGAGTACCG	GTCACTGGTCAACTCCAGCAC
Fabp4	NM_024406.2	ATCAGCGTAAATGGGGATTTGG	GTCTGCGGTGATTTCATCGAA
Acsl1	NM_007981.4	TCTTGGTGTACTACTACGACGAT	CGAGAACCTAAACAAGGACCATT
Dgat2	NM_026384.3	CGAGACACCATAGACTACTTGCT	GCGGTTCTTCAGGGTGACTG
Gpd1	NM_010271.3	CCATGTGGGTGTTTGAGGAAG	GCCCTGGCAGGTATTTAACATTC

### Western blot

Whole protein lysates were isolated using lysis buffer containing 20 mM HEPES, 1% Triton X-100, 150 mM NaCl, 1 mM EDTA, 2 mM Na_3_VO_4_, 10 mM NaF, and protease inhibitor cocktail (Quartett, Berlin, Germany). For nuclear and cytoplasmic fractionation, cells were harvested using a Subcellular Protein Fractionation Kit (Thermo Fisher Scientific) as per the manufacturer’s instructions. Protein concentrations were determined using a BCA Protein Assay kit (Thermo Fisher Scientific), and 10-μg samples were resolved by SDS-PAGE and transferred to a PVDF membrane (EMD Millipore). Membranes were blocked with 5% skim milk in TBS containing 0.1% Tween-20 (TBS-T) for 1 h and incubated in primary antibody diluted in 2% bovine serum albumin (BSA) containing TBS-T overnight at 4°C. Primary antibodies for phospho-p38, p38, phospho-GC receptor (GR), and GR were from Cell Signaling Technology (Danvers, MA, USA), and β-actin and lamin B1 were from Santa Cruz Biotechnology (Dallas, TX, USA). Membranes were washed with TBS-T three times for 10 min and incubated in anti-mouse or anti-rabbit HRP-conjugated secondary antibody (1:3000 dilution in TBS-T; Bio-Rad, Hercules, CA, USA) for 1 h at room temperature. Membranes were washed again three times for 10 min, developed using an EZ-Western Lumi Femto kit (DoGenBio, Seoul, Korea), and scanned using an ImageQuant LAS 4000 (GE Healthcare, Little Chalfont, UK).

### Animal experiments

BALB/c mice (female, 8 weeks old) were purchased from Orient Bio (Sungnam, South Korea) and housed at 21–23°C and 51–54% humidity with a 12-h light/dark cycle under pathogen-free conditions, and food and water were supplied *ad libitum*. Mice were injected with dexamethasone sodium phosphate (Dexa; 50 mg/kg) intraperitoneally once daily for 5 weeks, and T-MSC CM was injected intraperitoneally twice a week during the period of Dexa injection. The method of euthanasia was isoflurane inhalation followed by cervical dislocation, and all efforts were made to minimize suffering. All experiments were approved by the Animal Ethics Committee and Ewha Womans University School of Medicine (EUM20-014) and performed in accordance with relevant guidelines and regulations.

### Serum triglyceride and cholesterol measurement

Triglyceride and cholesterol levels in mouse serum after 5 weeks of treatments were determined by enzymatic methods and measured using an AU480 chemistry analyzer (Beckman Coulter, Brea, CA, USA).

### Hematoxylin and eosin staining

Isolated mouse femurs were fixed with 10% formalin solution, decalcified, paraffin-embedded, and sectioned at 4-μm thickness. Hematoxylin and eosin (H&E) staining was performed, and stained sections were scanned with an Aperio ScanScope slide scanner (Leica Biosystems, Wetzlar, Germany). Images were captured using CaseViewer software (3Dhistech, Budapest, Hungary), and bone marrow adiposity was analyzed using ImageJ software.

### Hematological analysis

Mouse blood samples were collected from the submandibular vein into EDTA-containing tubes (Golden Vac, Hermosillo, Mexico). Red blood cells, white blood cells, and platelets were counted using an Auto Hematology Analyzer (BC-2800Vet; Mindray, Shenzhen, China).

### Immunohistochemistry

Sectioned paraffin-embedded mouse femurs were hydrated and subjected to heat-induced antigen retrieval in Tris-EDTA buffer containing 10 mM Tris, 1 mM EDTA, and 0.05% Tween 20 (pH 9.0) for 20 min at 90–95°C. Peroxidase blocking for 30 min and protein blocking for 15 min were performed in a humid chamber using Dako reagents (Agilent, Sata Clara, CA, USA), after which anti-CD41 primary antibody incubation (1:500 diluted in PBS; Abcam) was performed overnight at 4°C. On the following day, specimens were incubated with a Dako LSAB2 System and streptavidin-HRP for 30 min each and then developed with 3,3’-diaminobenzidine solution. After counterstaining with hematoxylin, slides were dehydrated, mounted, and scanned for analysis.

### Megakaryocytic differentiation of K562 cells and coculture with OP9 adipocytes

The K562 human lymphoblast cell line was purchased from ATCC and cultured in RPMI medium supplemented with 10% FBS and antibiotics. Exponentially growing cells were induced for megakaryocytic differentiation by treating cells with 5 nM phorbol 12-myristate 13-acetate (PMA) for 48 h. For morphological analysis of cell differentiation, 3 × 10^4^ cells were collected using a Cytospin at 800 rpm for 5 min, fixed with methanol, and stained with Giemsa staining solution. For transwell co-culture, K562 cells were induced for differentiation in 0.4-μm pore size cell culture inserts and placed in 24-well plates containing OP9 adipocytes differentiated for 7 days, and co-cultures were incubated for 48 h.

### Statistical analysis

Statistical analyses were performed using GraphPad Prism 9.1.0 (GraphPad Software, La Jolla, CA, USA). Student’s *t*-tests were performed for comparisons of two groups, and one-way ANOVA in conjunction with Tukey’s post-hoc tests was performed for comparisons of three or more groups. Data are presented as mean ± standard error of the mean (SEM). *P*-values < 0.05 were considered statistically significant.

## Results

### T-MSC CM attenuates adipocyte differentiation from early stages

**Fig 1:**
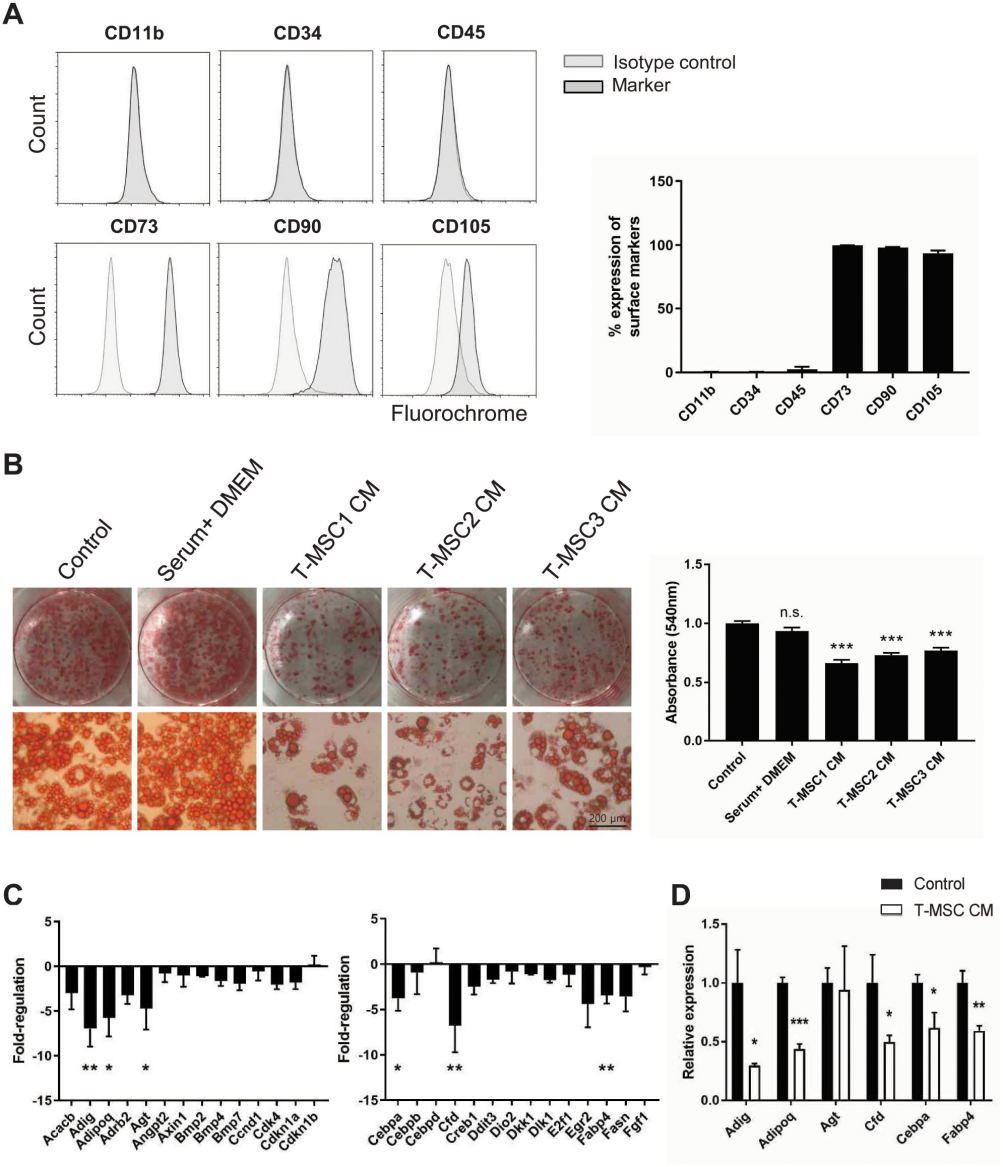
T-MSC CM attenuates adipocyte differentiation from early stages. A) Surface marker expression in T-MSCs from three different donors was examined by flow cytometry. Representative plots are shown and percentage expression of each marker is demonstrated (n = 3). B) 3T3-L1 preadipocytes underwent adipocyte differentiation induction in the presence or absence of T-MSC CM for 10 days. Lipid accumulation was determined using Oil Red O staining. Cells were treated with T-MSC CM from three different donors or serum-containing DMEM. Representative images are shown (200× original magnification). Quantification of Oil Red O staining measured at absorbance 540 nm. (C) Fold change in expression of genes involved in adipogenesis relative to control were determined in cells induced for differentiation for 3 days using an adipogenesis PCR array. Data were analyzed using one-way ANOVA (n = 3). (D) mRNA expression of differentially expressed genes was confirmed by RT-qPCR. Data are shown as mean ± SEM and were analyzed using Student’s *t*-tests (n = 3, *P < 0.05, **P < 0.01, ***P < 0.001).

We first examined the anti-adipogenic effects of T-MSC CM by treating cells during adipocyte differentiation with CM harvested from three different donors. To assess the MSC surface markers expression, flow cytometry analysis was performed and showed that cells expressed CD73, CD90, and CD105 but not CD11b, CD34, or CD45 (Fig 1A). Lipid accumulation was examined on differentiation day 10. T-MSC CM blocked lipid accumulation with minor donor-to-donor variation (Fig 1B). This was not observed in cells treated with serum-containing media, demonstrating the paracrine anti-adipogenic effects of T-MSCs. Next, we performed adipogenesis PCR array using transcripts isolated at early stages of differentiation day 3. T-MSC CM tended to reduce the expression of most genes involved in adipogenesis, with significant changes in the expression of Adig, Adipoq, Agt, Cebpa, Cfd, and Fabp4 (Fig 1C). Real-time quantitative PCR analysis confirmed that all selected genes except Agt were downregulated by T-MSC CM (Fig 1D).

### T-MSC CM selectively inhibits GC signaling

**Fig 2:**
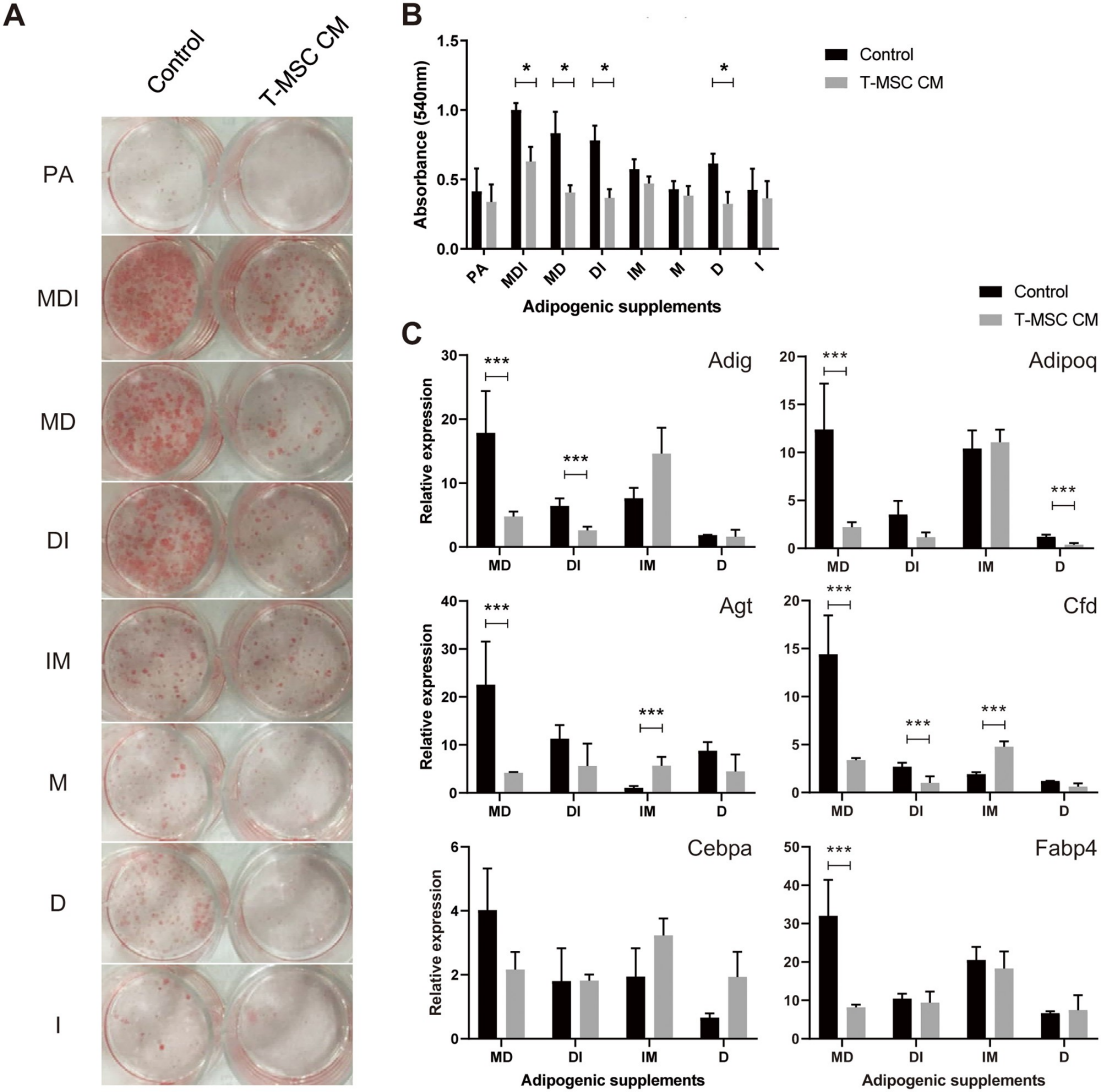
T-MSC CM selectively inhibits GC signaling. Adipocyte differentiation was induced using adipogenic factors individually or in combinations with or without T-MSC CM. (A) Photomicrograph of adipocyte differentiation taken after Oil Red O staining on differentiation day 10. (B) Quantification of Oil Red O staining measured at absorbance 540 nm. (C) mRNA expression of the adipogenic genes Adig, Adipoq, Agt, Cfd, Cebpa, and Fabp4 on differentiation day 3 was determined using RT-qPCR. Data are presented as mean ± SEM and were analyzed using Student’s *t*-tests (n = 3, *P < 0.05, ***P < 0.001). PA, preadipocyte; M, IBMX; D, dexamethasone; I, insulin.

To identify which adipogenic pathway is affected by T-MSC CM, we examined the effects of T-MSC CM in the presence of individual adipogenic factors (IBMX, M; dexamethasone, D; and insulin, I) or cocktails of different factors (MDI, MD, DI, and IM). After cells were induced for differentiation, lipid accumulation was examined on day 10. T-MSC CM had an anti-adipogenic effect only when dexamethasone was present (MDI, MD, DI, and D; Fig 2A and 2B). Highly downregulated gene expression of Adig, Adipoq, Agt, and Fabp4 was observed on day 3 in the presence of MD (Fig 2C). T-MSC CM had limited effects on gene expression in the presence of other combinations of factors, likely due to the reduced adipogenic potency of incomplete cocktails. Agt and Cfd expression were increased by T-MSC CM in the presence of IM.

### T-MSC CM enhances GR phosphorylation and inhibits nuclear translocation

**Fig 3:**
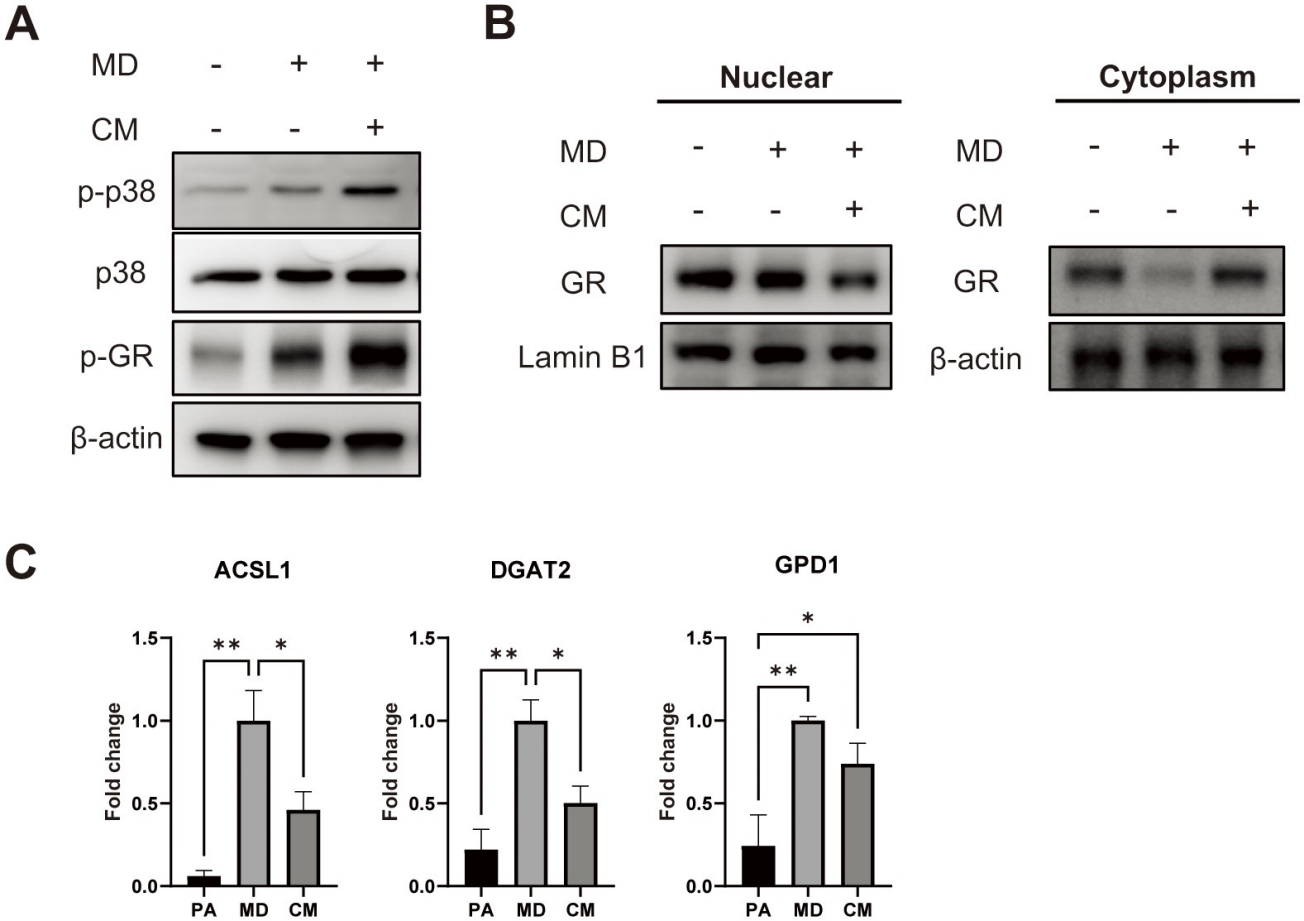
T-MSC CM enhances GR phosphorylation and inhibits its nuclear translocation. (A) Representative Western blots of whole cell lysates treated with MD for 1 h in the presence or absence of T-MSC CM. (B) After 8 h of treatment, cells were divided into nuclear and cytoplasmic fractions, and GR levels were examined by Western blots. (C) mRNA expression of GR-responsive lipid metabolism genes Acsl1, Dgat2, and Gpd1 was determined by RT-qPCR. Data are presented as mean ± SEM and were analyzed using one-way ANOVA (n = 3, *P < 0.05, **P < 0.01).

Next, we examined the effects of T-MSC CM treatment on GC signaling. p38 mitogen-activated protein kinase (p38)-induced GR phosphorylation is one mechanism by which GC signaling is reduced via inhibition of GR translocation [17,18]. We found that T-MSC CM promoted phosphorylation of p38 and GRs (Fig 3A). Reduced GR translocation was also determined by nuclear and cytoplasmic fractionation (Fig 3B). When we examined the expression of GR-responsive lipid metabolism genes, we found that acyl-coA synthetase long-chain family member 1 (ACSL1), diacylglycerol O-acyltransferase 2 (DGAT2), and glycerol-3-phosphate dehydrogenase 1 (GPD1) expression was inhibited by T-MSC CM in the presence of MD (Fig 3C).

### T-MSC CM reduces GC-induced visceral and bone marrow adiposity

**Fig 4:**
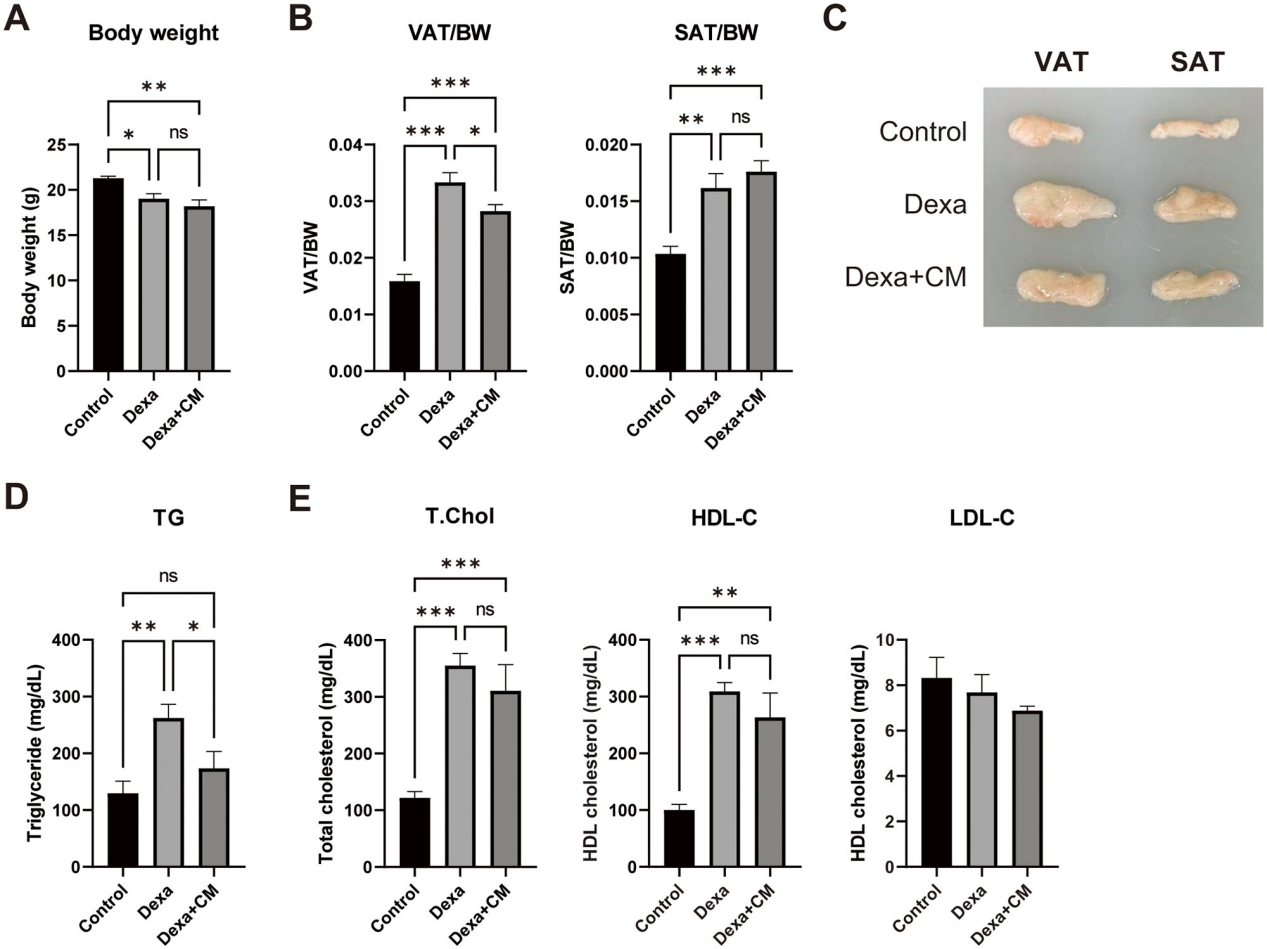
T-MSC CM reduces GC-induced visceral adiposity. BALB/c female mice were intraperitoneally injected with Dexa once daily for 5 weeks. T-MSC CM was injected twice a week. (A) Body weight was measured on the day of sacrifice. (B) Visceral (VAT) and subcutaneous (SAT) adipose tissue were harvested, and measured weights were normalized to body weight. (C) Photomicrograph of VAT and SAT. Serum levels of (D) triglycerides and (E) cholesterols were measured. Data are presented as mean ± SEM and were analyzed using one-way ANOVA (n = 5; ns, not significant; *P < 0.05, **P < 0.01).

We further examined whether T-MSC CM can ameliorate GC-induced adipogenesis *in vivo* by injecting mice with Dexa, a synthetic GC, for 5 weeks. After 5 weeks of high-dose Dexa treatment, mouse body weight was significantly reduced compared with control mice (Fig 4A). Next, mice were sacrificed, and visceral and subcutaneous adipose tissue were weighed and normalized to body weight. We found that Dexa treatment enhanced both visceral and subcutaneous adipose tissue deposition, whereas T-MSC CM supplementation reduced visceral fat mass (Fig 4B and 4C). Dexa treatment also increased serum triglycerides levels, but this was blocked by Dexa + CM treatment (Fig 4D). Total cholesterol levels were increased in both Dexa and Dexa + CM groups compared with the control group mainly due to an increase in high-density lipoprotein cholesterol, whereas low-density lipoprotein cholesterol levels were similar among groups (Fig 4E).

**Fig 5:**
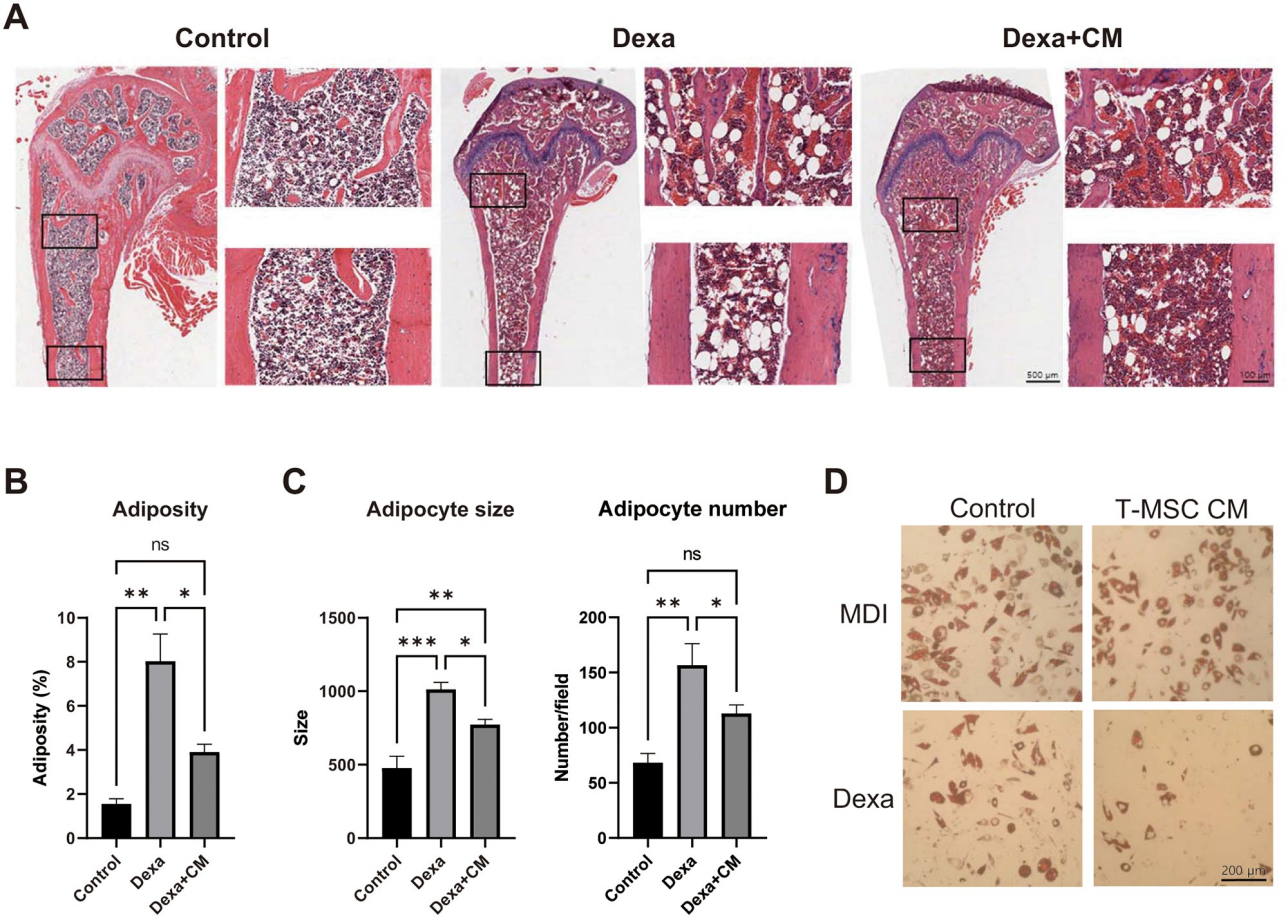
T-MSC CM reduces GC-induced bone marrow adiposity. (A) Representative images of H&E staining from proximal mouse femurs (30× and 200× original magnification). (B) Bone marrow adiposity (%) and (C) adipocyte number and size were analyzed using ImageJ software. (D) OP9 mouse bone marrow stromal cells were cultured and treated with adipogenic cocktail or Dexa only, and lipid accumulation was determined by Oil Red O staining. Data are presented as mean ± SEM and were analyzed using one-way ANOVA (n = 5; ns, not significant; *P < 0.05, **P < 0.01).

As previous studies show that GC signal transduction plays a role in the development of bone marrow adipose tissue (BMAT) [19–21], we examined whether T-MSC CM could modulate BMAT accumulation. When we examined mouse bone marrow adiposity in femur metaphysis and diaphysis using H&E staining, we found that Dexa treatment significantly increased BMAT accumulation, but this was blocked by T-MSC CM treatment (Fig 5A and 5B). We also analyzed the size and number of bone marrow adipocytes and found that T-MSC CM protected against bone marrow adipocyte hypertrophy and hyperplasia (Fig 5C). In addition, T-MSC CM inhibited bone marrow adipocyte differentiation in the presence of MDI using an OP9 mouse bone marrow stromal cell line (Fig 5D).

### T-MSC CM promotes platelet production

**Fig 6:**
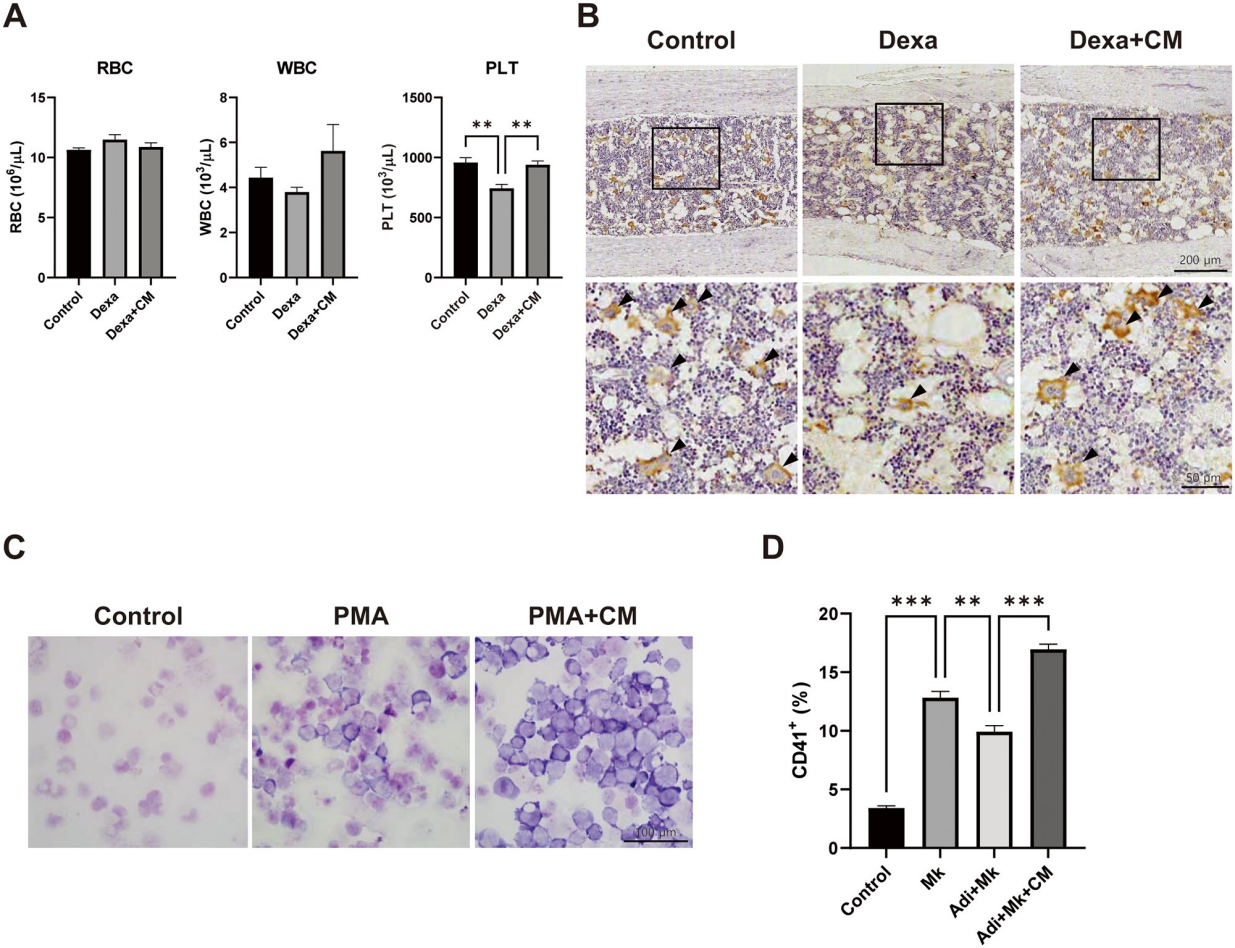
T-MSC CM promotes platelet production. (A) Red blood cells (RBCs), white blood cells (WBCs), and platelets (PLTs) in mouse peripheral blood were counted using an auto hematology analyzer on the day of sacrifice. Data are presented as mean ± SEM and were analyzed using one-way ANOVA (n = 5, **P < 0.01). (B) Immunohistochemical analysis of megakaryocytes in mouse bone marrow. Representative images with a higher magnification in the lower panel (200× original magnification). (C) K562 human lymphoblasts were induced for megakaryocytic differentiation in the presence or absence of T-MSC CM. Representative images of cells with Giemsa staining (200× original magnification). (D) Megakaryocyte differentiation was induced with or without T-MSC CM, and the effects of OP9 adipocyte co-culture were examined. Expression levels of megakaryocyte marker CD41 were determined using flow cytometry. Data are presented as mean ± SEM and were analyzed using one-way ANOVA (n = 3; *P < 0.05, ***P < 0.001).

Given that bone marrow is the primary site of hematopoiesis and that bone marrow adipocytes are known negative regulators of hematopoiesis [22], we performed a complete blood count with mouse peripheral blood. Numbers of red and white blood cells did not significantly differ among groups. Interestingly, platelet numbers were reduced by Dexa treatment, but this was blocked by T-MSC CM treatment (Fig 6A). When we performed histological analysis of bone marrow megakaryocytes, we found that Dexa treatment reduced the number of megakaryocytes in bone marrow, but this was blocked by T-MSC CM treatment (Fig 6B). Next, we examined the effects of T-MSC CM on megakaryocyte differentiation using the K562 human lymphoblast cell line. PMA induced megakaryocytic differentiation and promoted cell differentiation in the presence of T-MSC CM as shown by an increase in cell size and nuclear-to-cytoplasm ratio (Fig 6C). Finally, we investigated the effects of adipocytes on megakaryocytic differentiation as evidenced by surface expression of megakaryocyte markers CD41 in a K562 and OP9 co-culture system. We found that PMA-induced CD41 expression was reduced in co-culture with mature adipocytes, and was further enhanced by T-MSC CM treatment (Fig 6D).

## Discussion

We found that T-MSC CM inhibits GC-induced adipocyte differentiation by promoting phosphorylation of p38 and GRs, thereby reducing GC signal transduction. In addition, T-MSC CM reduced visceral and bone marrow adiposity, and increased platelet production in Dexa-injected BALB/c mice.

GCs signal through GRs, which reside in the cytoplasm, translocate into the nucleus upon ligand binding, and then activate or repress transcription. The transcriptional activities of GRs are diverse due to the diversity in GR response elements, GR isoforms, and post-translational modifications to GRs [23]. GR phosphorylation by p38 inhibits the nuclear translocation of GRs, which contributes to GC insensitivity in patients with asthma [18]. In the present study, we demonstrated that T-MSC CM inhibits GC signaling by enhancing the phosphorylation of p38 and GRs.

Synthetic GCs are widely prescribed for relieving inflammatory conditions of the skin, eye, and respiratory system. However, long-term high-dose GC therapy can cause adverse side effects including skin atrophy, osteoporosis, Cushing’s syndrome, and diabetes, mainly via transactivation of signaling [24]. As the anti-inflammatory effects of GCs are mediated by transrepression, the development of novel drugs that can minimize side effects while preserving anti-inflammatory effects is needed. MSCs are reported to possess immunoregulatory properties in many diseases [25,26], which are achieved via the secretion of anti-inflammatory cytokines. Future studies should verify whether T-MSC CM exerts anti-inflammatory effects while inhibiting the side effects of GCs using an inflammatory disease mouse model. This would provide a framework for developing novel therapeutics to regulate inflammation when synthetic GCs do not function properly.

GC-induced osteoporosis is a concern when GC treatment is used long-term, as bone loss and an increased rate of osteoporotic fractures are observed in patients treated with GCs for more than 6 months [27,28]. BMAT expansion could be an underlying mechanism of GC-induced osteoporosis, as studies demonstrate a close relationship between BMAT and osteoporosis [29,30]. BMAT has characteristics that are distinguishable from those of white or brown adipose tissue considering morphology, gene expression, and fatty acid composition [31–33]. Although signals involved in BMAT development and function are poorly understood, Cawthorn et al. reported that GC signaling is a driving factor of BMAT expansion [20]. In the present study, we observed increased BMAT after chronic Dexa treatment, which was attenuated by the inhibition of GC signaling via T-MSC CM. These findings suggest that MSC CM could be used therapeutically for GC-induced osteoporosis and visceral obesity.

BMAT is also involved in hematopoiesis. BMAT increases with age and occupies 50–70% of the bone marrow cavity in adults [31]. As BMAT increases, hematopoietic cellularity decreases along with numbers of red and white blood cells in circulation [34]. Naveiras et al. reported that numbers of hematopoietic progenitor cells and their colony-forming units are negatively linked to bone marrow adiposity and that bone marrow regeneration is accelerated in fatless transgenic mice [22]. These results suggest that regulation of BMAT using T-MSC CM could be employed as a therapeutic strategy to enhance bone marrow reconstitution. Our research group is currently investigating whether T-MSCs enhance bone marrow transplantation efficacy. In a series of experiments, we show that faster bone marrow reconstitution is accompanied by reduced bone marrow adiposity [35,36]. Further research identifying effector molecules that can enhance bone marrow reconstitution via inhibition of BMAT expansion is required.

We also found that T-MSC CM protected Dexa-injected mice from platelet reduction. Immunohistochemical analysis of bone marrow demonstrated that T-MSC CM increased numbers of megakaryocytes, and enhanced megakaryocyte differentiation was determined *in vitro*. We further investigated whether megakaryocyte differentiation is affected by adipocytes using a co-culture system and observe a significant inhibition in the presence of adipocytes. This suggests that T-MSC CM may directly and/or indirectly regulate megakaryocyte differentiation, as we recently reported that T-MSC CM promotes megakaryopoiesis by secretion of placental growth factor [37]. It would be premature, however, to conclude the role of BMAT in platelet production. Given that our observations were limited to megakaryocyte differentiation *in vitro*, more detailed research is required to elucidate the roles of BMAT in megakaryocyte maturation and platelet production *in vitro* and *in vivo*.

In summary, we show that T-MSC CM inhibited adipocyte differentiation by negatively regulating GC signaling. T-MSC CM induced phosphorylation of p38 and GR, thereby inhibiting nuclear translocation and subsequent transcription. Inhibition of GC signaling attenuated fat accumulation in the abdominal and bone marrow cavities in mice. Further work is required to identify the effector molecules of T-MSC CM that attenuate adipocyte differentiation and to determine the significance of BMAT reduction in the promotion of bone marrow function. Such research will provide new insights into the therapeutic potential of T-MSC CM to reduce obesity and the prevalence of obesity-related diseases.

## Supporting information

S1 Raw images (PDF)
