# Supplementary material for: Conditioned medium from human tonsil-derived mesenchymal stem cells inhibits glucocorticoid-induced adipocyte differentiation
Source: PLoS One. 2022 Jun 1;17(6):e0266857. doi: 10.1371/journal.pone.0266857 (PMC9159628; doi:10.1371/journal.pone.0266857)
Supplement: S1 Raw images — (PDF) [file pone.0266857.s001.pdf]

Fig 3A

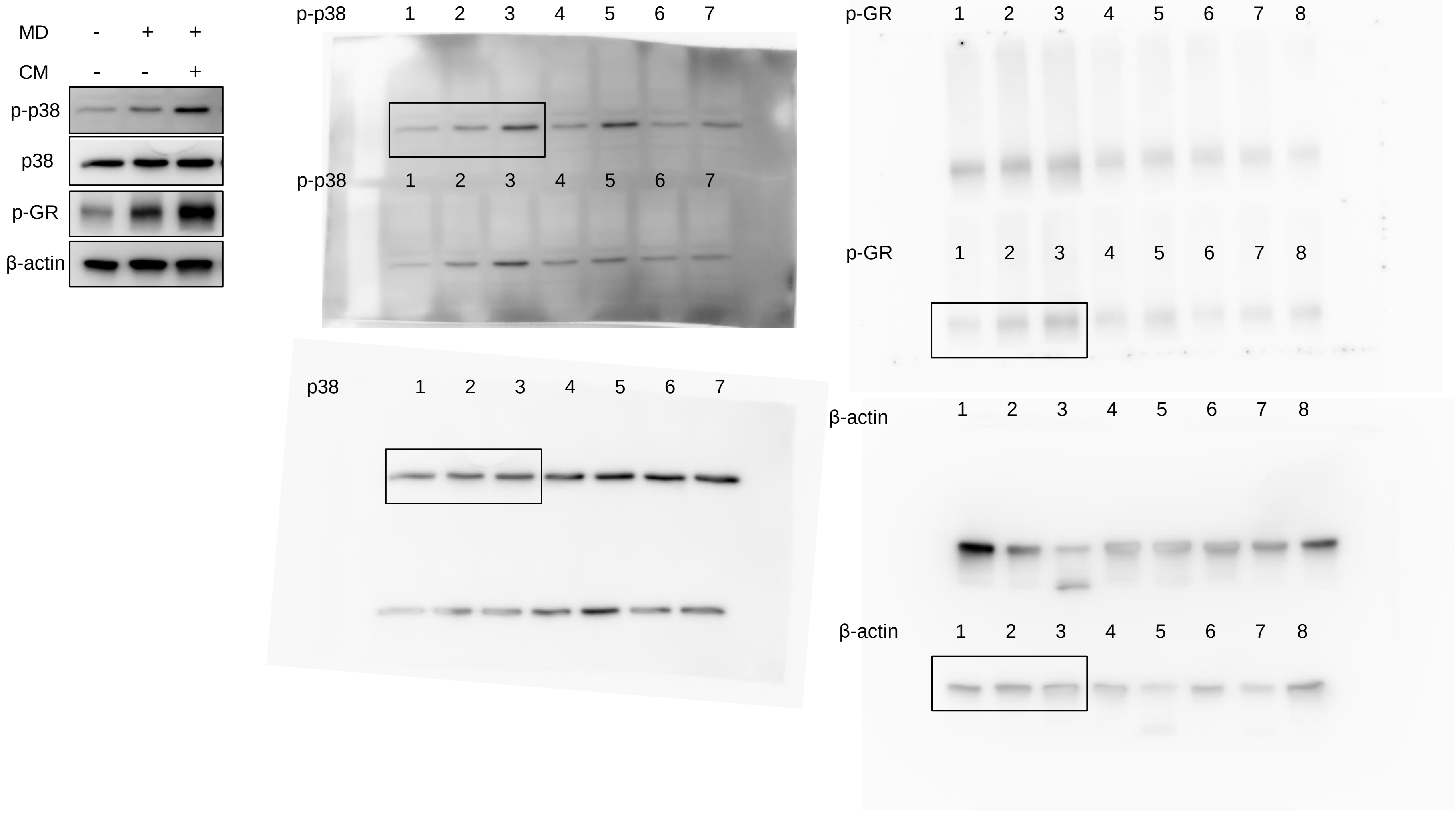

1. Preadipocytes
2. MD treatment 1h
3. MD+CM treatment 1h
4. MD treatment 2h
5. MD+CM treatment 2h
6. MD treatment 4h
7. MD+CM treatment 4h

1. Preadipocytes
2. MD treatment 1h
3. MD+CM treatment 1h
4. MD treatment 8h
5. MD+CM treatment 8h
6. MD+rhIGFBP4 8h
7. MD treatment 24h
8. MD+CM treatment 24h

Fig 3B

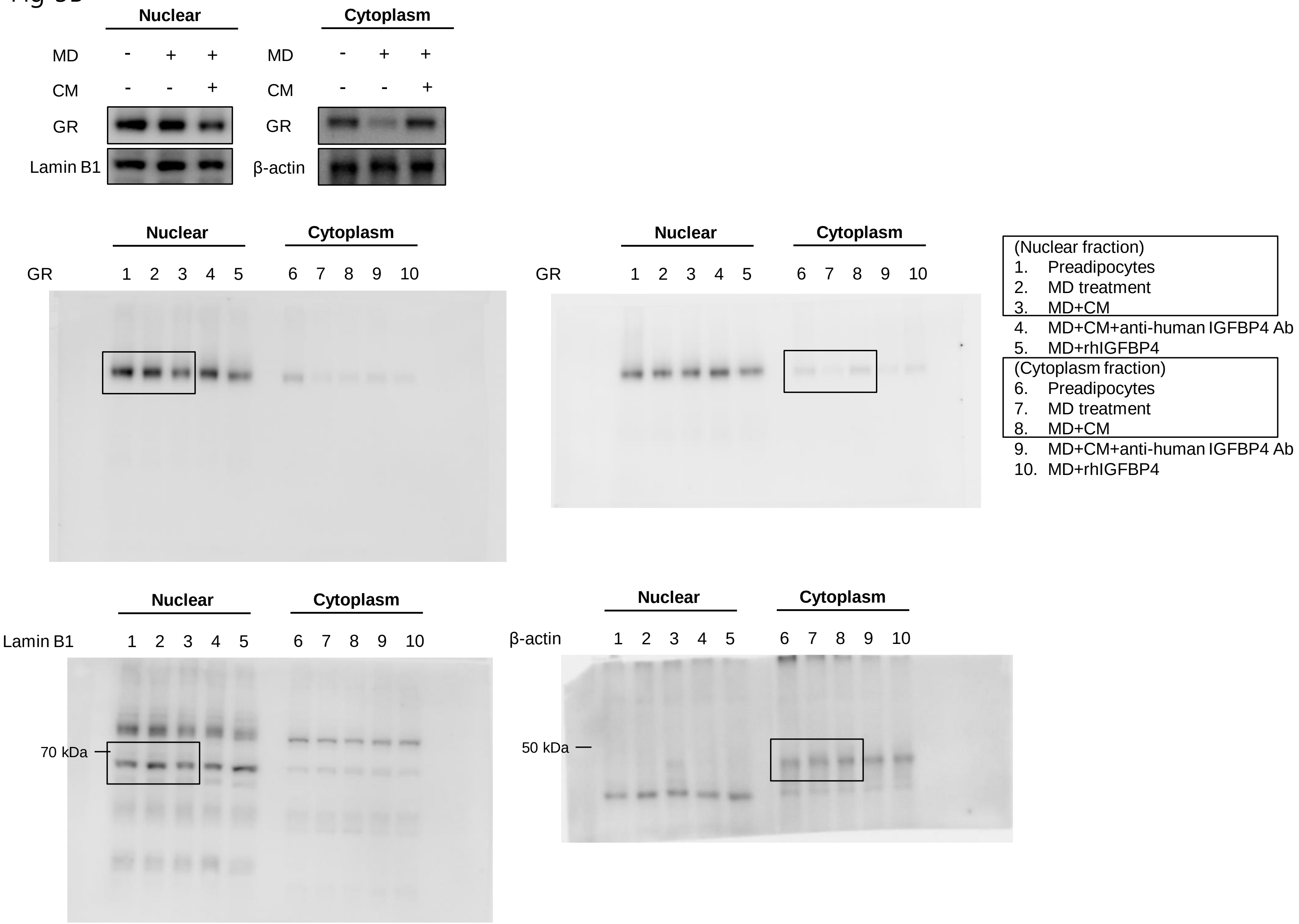
Nuclear
Cytoplasm
MD - + +
CM - - +
GR
Lamin B1
MD - + +
CM - - +
GR
β-actin
Nuclear
Cytoplasm
GR 1 2 3 4 5 6 7 8 9 10
Nuclear
Cytoplasm
GR 1 2 3 4 5 6 7 8 9 10
(Nuclear fraction)
1. Preadipocytes
2. MD treatment
3. MD+CM
4. MD+CM+anti-human IGFBP4 Ab
5. MD+rhIGFBP4
(Cytoplasm fraction)
6. Preadipocytes
7. MD treatment
8. MD+CM
9. MD+CM+anti-human IGFBP4 Ab
10. MD+rhIGFBP4
Nuclear
Cytoplasm
Lamin B1 1 2 3 4 5 6 7 8 9 10
70 kDa
Nuclear
Cytoplasm
β-actin 1 2 3 4 5 6 7 8 9 10
50 kDa
